# Experience with Selective Testing of Plasmodium Parasites in Swiss Blood Donors

**DOI:** 10.3390/pathogens15060614

**Published:** 2026-06-08

**Authors:** Mauro Serricchio, Muriel Fragnière, Jochen Gottschalk, Caroline Tinguely, Christoph Niederhauser

**Affiliations:** 1 Interregional Blood Transfusion SRC, 3008 Bern, Switzerlandcaroline.tinguely@itransfusion.ch (C.T.); christoph.niederhauser@itransfusion.ch (C.N.); 2 Institute for Infectious Diseases, University of Bern, 3001 Bern, Switzerland; 3 Blood Transfusion Service SRC Zürich, 8952 Schlieren, Switzerland

**Keywords:** malaria, blood transfusion, blood donor screening, *Plasmodium*, EIA, Switzerland

## Abstract

Transmission of malaria by blood transfusion is rare in non-endemic countries but can lead to serious complications in blood recipients. Increasing travel to and immigration from regions at risk for tropical diseases poses a challenge to blood donation services, which are striving to reduce the number of blood donor deferrals while ensuring a high level of blood safety. National guidelines of the Blood Transfusion Service of the Swiss Red Cross demand that donors at risk are serologically tested for malaria antibodies. Here, we summarize the numbers of malaria tests performed and the results obtained since the introduction of mandatory testing in Switzerland in 2007. From malaria-positive donors, information on travels to endemic areas and place of origin, or if malaria symptoms were experienced and if prophylaxis was taken, was requested in a post-donation questionnaire.

## 1. Introduction

Malaria is a serious and potentially life-threatening disease. Globally, in 2024, the number of malaria cases was ~282 million, and the number of deaths was estimated at 610,000 [1], with millions of people living in risk-areas worldwide. Malaria is caused by protozoan parasites of the genus *Plasmodium*, which are transmitted to humans through the bites of infected female *Anopheles* mosquitoes. There are several species of *Plasmodium* that routinely infect humans: *P. falciparum*, *P. vivax*, *P. ovale* and *P. malariae* [1,2]. The life cycle of the malaria parasite requires two hosts: the *Anopheles* mosquito and the human. During a blood meal, an infected female *Anopheles* mosquito injects sporozoites into the human host. These sporozoites invade liver cells and develop into schizonts [3]. After this initial replication phase in the liver, the parasites multiply asexually within red blood cells. It is the blood stage of the parasites that causes clinical symptoms of the disease. Malaria symptoms typically include fever, chills, sweating, headaches, nausea, and fatigue, and if left untreated, the disease can lead to severe complications, including organ failure and death [4].

Malaria is primarily found in tropical and subtropical regions, including parts of sub-Saharan Africa, South Asia, and South America. Efforts to control malaria have focused on insecticide-treated bed nets, antimalarial medications, and efforts to eliminate mosquito breeding sites, but the disease remains a major global health challenge. Despite advances in prevention and treatment, malaria continues to cause significant morbidity and mortality, especially in young children and pregnant women [1]. The parasite’s genetic adaptability and its tendency to develop resistance to drugs have long been a problem in the fight against malaria [5].

Malaria can be transmitted by transfusion of blood components, especially red blood cells, and transfusion-transmitted malaria (TTM) still poses a risk for transfusion services worldwide [6]. So-called “semi-immune” donors pose the highest risk for transmitting malaria, because they carry undetectable levels of parasites in their blood [7,8]. These asymptomatic carriers develop a partial immunity over time through repeated exposure to the parasite. They are usually protected from developing severe symptoms but can transmit plasmodium via blood products [7,8].

In Europe, malaria poses a big problem, because (i) a growing number of potential blood donors have a background from a malaria-endemic area, and (ii) many recipients of blood transfusions are immunocompromised. Blood banks employ different strategies to reduce the risk of malaria transmission. Commonly, donors at risk of malaria are excluded from donating for a period of 6 months to 3 years, depending on the deferral policy of the individual country. This deferral strategy is reinforced by selective malaria screening programs in several European countries. Historically, diagnostic tools to detect plasmodium parasites included blood film examination, antibody detection (IFA, ELISA), antigen detection, or nucleic acid amplification by PCR, TMA or other technologies. Direct parasite detection techniques lack sensitivity to reliably identify contaminated blood products. Most countries that have implemented a selective testing approach for donors at risk use antibody screening tests.

This approach is also mandatory in Switzerland since 2007. The precautions implemented seem to be safe, as the last transfusion-transmitted malaria case was reported in 1999 [7]. Here, we summarize *Plasmodium* spp. testing in Switzerland since its implementation in 2007, report case numbers, countries of origin and place of infection in travel-related malaria cases.

## 2. Materials and Methods

The national guidelines published by Blood Transfusion Swiss Red Cross for donors at risk for malaria propose that donors travelling to regions at risk for malaria are deferred from donating blood for 6 months. Donors suffering from fever of unknown origin or other malaria-typical symptoms, those with previous malaria anamnesis or those born or staying in a region at risk for malaria for longer than 6 months can donate blood after a negative *Plasmodium* spp. antibody test performed ≥4 months after their return or recovery. From 2007 until 2011, either an EIA malaria test (Bio-Rad, Cressier, Switzerland) or, in part, an in-house assay was used to detect anti-malaria antibodies. From 2011 until 2023, all donors were screened with an EIA malaria test (Bio-Rad, Cressier, Switzerland). From December 2023 until October 2024, samples were sent directly to the national reference laboratory for analysis (ELISA, IFAT) since no IVDR Class D compliant test was available in Switzerland. Starting November 2024, donors were screened with an EIA malaria test from DiaPro (DiaPro, Milan, Italy).

From donors testing positive for malaria, additional data concerning place of birth, length of stay in region at risk, age, gender, intake of prophylaxis, diagnosis and possible treatments was collected in a post-donation questionnaire.

## 3. Results

### 3.1. Confirmed Malaria-Positive Blood Donations 2007–2025

**Figure 1:**
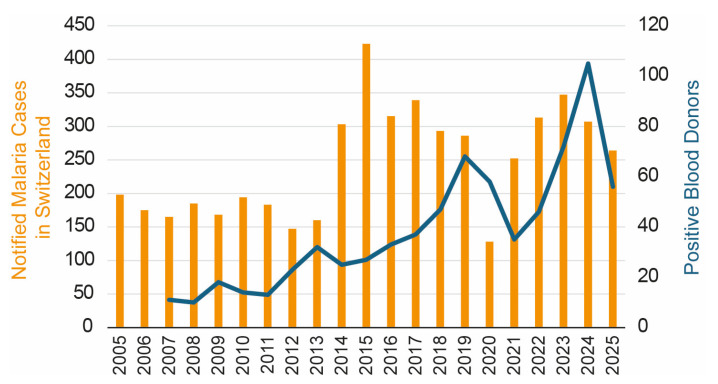
Notified malaria cases in Switzerland and number of positive blood donors. Yearly notified case numbers in the period of 2005–2025 are shown in orange columns; yearly positive blood donations detected in the period of 2007–2025 are depicted with the blue line.

According to the national guidelines issued by the Blood Transfusion Service of the Swiss Red Cross, blood donated at one of the regional blood transfusion services is selectively screened for the presence of anti-*Plasmodium* spp. antibodies by enzyme immunoassays (EIA). The screening was introduced as mandatory in 2007 for blood donors returning from an endemic area or for immigrants from endemic areas. In the 18 years since introduction of malaria testing, a total of 30956 blood donations were screened for malaria. Donations tested for malaria amount to 0.53% of all the 5.85 Mio blood donations during this time. Of the donations tested for malaria, 28,296 were negative, 2430 (7.8%) were reactive, and 230 (0.7%) were in the grey zone of the assay used. Reactive and borderline reactive blood components were removed from the blood supply. Of the 2660 initial positive or borderline donations, 735 (2.4%) were confirmed positive by the Swiss Tropical and Public Health Institute. Yearly blood donor case numbers showed a steady increase between 2007 and 2019, with a sharp drop in detected malaria cases in the year 2021 (Figure 1). This drop, caused by travel limitations during the SARS-CoV-2 pandemic, was accompanied by a rapid recovery in the following years. In 2023, malaria cases exceeded pre-pandemic levels and peaked in 2024. Numbers of malaria-positive blood donors do not correlate with the total number of malaria cases reported by the federal office of public health (Figure 1). Notified malaria cases in Switzerland remained below 200 cases per year in the years 2005–2013, with a rapid increase in 2014 and 2015 (Figure 1). Ever since, the number of notified malaria cases remained elevated, except for the COVID-19-related drop in 2020.

### 3.2. Post-Donation Information of Confirmed Malaria-Positive Donations 2007–2025

In a post-donation questionnaire sent to the doctors of the respective blood donation service, information on the country of origin or past travels to endemic areas was requested from malaria-positive donors. We were able to evaluate 433 questionnaires, which accounted for 59% of all confirmed positive donations. For the remaining confirmed positive donations, no status could be documented. Of the malaria-positive donors that provided information, 355 originated from an endemic country: 302 from Africa, 35 from Asia, and 18 from Soutth America (Table 1). A more in-depth look at the location of origin revealed that most malaria-positive donors originated from Cameroon, Eritrea, Ivory Coast, Congo, and Nigeria (Table 2). In contrast, 78 donors were diagnosed malaria-positive after having travelled to malaria-endemic areas: 64 with a travel history to Africa, 7 to Asia, and 3 to South America (Table 1). While in the period from 2007 to 2015 only four travel-related malaria cases were reported [9], 74 travel-associated positive donations were found in the period of 2016–2025.

### 3.3. Post-Donation Information on Symptoms and Prophylaxis in Positive Donations from 2007 to 2025

The post-donation questionnaire also included questions about whether prophylaxis was taken or if symptoms were experienced. Of donors that were infected while travelling, only 10 reported taking prophylaxis (Atovaquon, Mefloquine, Chloroquine, Malarone), while 28 did not take any preventive medicine. Eight travelling donors who did not take prophylaxis do not recall having suffered from malaria symptoms, and two donors had malaria symptoms despite taking prophylaxis. Of donors originating from an endemic country, 62 (without taking prophylaxis) and 6 (with taking prophylaxis) did not recall having malaria symptoms in their youth.

## 4. Discussion

Transfusion-transmission of malaria is a major concern in non-endemic countries, especially since changes in human behavior like extensive travels to malaria-endemic countries and migration from malaria-endemic countries have increased. The issue with transfusion-transmitted malaria is multi-layered. On one hand, plasmodium parasites survive in whole blood when stored at 4 °C up to 28 days [10], although extended storage affects the viability of the parasites. On the other hand, only very few remnant parasites are needed to infect transfusion recipients. Three TTM cases due to transfusion of platelet concentrates in Canada suggest that very small numbers of infected erythrocytes are sufficient for transmission of malaria [8].

A major issue for blood donation services in non-endemic countries is the effort to reduce donor deferrals while maintaining a high level of blood safety. Different countries adopt varied strategies to address this problem, including temporary deferrals, permanent deferrals and selective testing. To identify the donors at risk, preanalytical approaches rely on the specific questionnaire and the truthful answers given within. Many countries including Switzerland cannot afford to defer all donors at-risk, since a significant proportion of potential blood donors would be lost. As a non-endemic country with few imported infections, Switzerland has implemented the same procedure as other non-endemic countries like Canada, France, the UK and others [11,12,13,14]. This procedure assumes that positive donors would be most efficiently identified by a specific donor questionnaire followed by selective serological testing of donors at risk. With this strategy, Switzerland had no reported transfusion-transmission since 1999 [7].

It is important to differentiate between donors who have lived and grown up in endemic regions and short-terms travelers. Most of the donors at risk are short term travelers, and those are mostly antibody negative. The proportion of donors who tested positive for malaria is significantly higher among those who have lived in endemic areas for an extended period or grew up there. By including specific questions in the questionnaire and distinguishing between travelers returning from endemic countries and individuals originating from high-risk areas, the destruction of blood products and the deferral of blood donors could potentially be further optimized.

The use of screening methods is of paramount importance to prevent donations of potentially malaria-transmitting blood. In this regard, serology appears to offer these guarantees. In fact, in a systematic review of transfusion-transmitted-malaria (TTM) in a non-endemic area, only 100 cases of TTM were reported and thirty-eight of these occurred in Europe [15]. The main challenge is to develop an optimal screening algorithm that ensures the safety and quality of blood components while avoiding a loss of potential donors. The first essential step in this algorithm, which includes an initial structured questionnaire to assess the risk for malaria infection and subsequent immunological testing, should be maintained. Some plasmodium species (*P. vivax* and *P. ovale*) can form hypnozoites able to evade the host immune response [2]. A critical window is the period between activation—that is, the onset of blood-borne stages—the onset of symptoms, and the formation of antibodies. In theory, a blood sample taken during this period could contain the parasite but not yet show any antibodies.

However, new strategies should be explored to avoid the loss of potential donors with low-incidence red cell antigens, which are rare in Caucasians but common in people from Africa, Asia, or Latin America [16,17,18]. If the origin of these donors is known, they are broadly genotyped for specific red cell antigens of the corresponding populations. To ensure that the corresponding patient group receives the best possible care, including blood products compatible with their genotype, a combined approach involving serological testing and molecular techniques is beneficial. In this case it could be possible to transfuse RBC from anti-*Plasmodium*-positive blood donors—who are, however, PCR or TMA negative—to these patients with rare African, Asian or Latin American blood group antigens. This decision is a risk-based approach and must, of course, always rest with the treating physician.

In summary, the algorithm currently in use appears to be very safe regarding the transfusion of blood products to patients. For certain patients from African, Asian or Latin American populations, the use of supplementary molecular tests could potentially improve the supply of blood with specific red cell antigens.

## Figures and Tables

**Table 1:** Number of donations with *Plasmodium* spp. antibody-confirmed blood. The number of donors originating from malaria-endemic countries and number of donors with travel history to endemic countries are shown. Data from 2007 to 2025 are included.

	# With Origin in Endemic Country (%)	# Travel-Related Infection (%)
Africa	302 (85)	64 (82)
Asia	35 (10)	7 (9)
South America	18 (5)	3 (4)
Unknown		4 (5)
Total	355 (100)	78 (100)

**Table 2:** Countries of origin of malaria-positive blood donors in Switzerland, 2007–2025. “Others” represent countries with ≤2 (Africa) or ≤1 (Asia, South America) positive cases.

	Country of Origin	# Of Positive Donations	% Male
Africa	Cameroon	63	35
	Eritrea	34	88
	Ivory Coast	35	42
	Congo	21	71
	Nigeria	13	62
	Togo	21	70
	Benin	7	100
	Senegal	6	100
	Rwanda	5	40
	Somalia	5	80
	Burkina Faso	4	50
	Mali	4	100
	Guinea	3	100
	South Africa	3	67
	Others	78	
Asia	India	10	50
	Pakistan	4	100
	Sri Lanka	2	50
	Others	19	
South America	Brazil	6	40
	Columbia	2	0
	others	10	

## Data Availability

The original contributions presented in this study are included in the article. Further inquiries can be directed to the corresponding author.

## Abbreviations

The following abbreviations are used in this manuscript:

EIA	Enzyme Immunoassay
NAT	Nucleic Acid Test
ELISA	Enzyme-Linked-Immunosorbent-Assay
IFAT	Immuno-Fluorescence-Antibody-Test
